# Lack of correlation between reaction speed and analytical sensitivity in isothermal amplification reveals the value of digital methods for optimization: validation using digital real-time RT-LAMP

**DOI:** 10.1093/nar/gkv877

**Published:** 2015-09-10

**Authors:** Eugenia M. Khorosheva, Mikhail A. Karymov, David A. Selck, Rustem F. Ismagilov

**Affiliations:** Division of Chemistry and Chemical Engineering, California Institute of Technology, 1200 East California Blvd., Pasadena, CA 91125, USA

## Abstract

In this paper, we asked if it is possible to identify the best primers and reaction conditions based on improvements in reaction speed when optimizing isothermal reactions. We used digital single-molecule, real-time analyses of both speed and efficiency of isothermal amplification reactions, which revealed that improvements in the speed of isothermal amplification reactions did not always correlate with improvements in digital efficiency (the fraction of molecules that amplify) or with analytical sensitivity. However, we observed that the speeds of amplification for single-molecule (in a digital device) and multi-molecule (e.g. in a PCR well plate) formats always correlated for the same conditions. Also, digital efficiency correlated with the analytical sensitivity of the same reaction performed in a multi-molecule format. Our finding was supported experimentally with examples of primer design, the use or exclusion of loop primers in different combinations, and the use of different enzyme mixtures in one-step reverse-transcription loop-mediated amplification (RT-LAMP). Our results show that measuring the digital efficiency of amplification of single-template molecules allows quick, reliable comparisons of the analytical sensitivity of reactions under any two tested conditions, independent of the speeds of the isothermal amplification reactions.

## INTRODUCTION

The detection and quantification of nucleic acids using quantitative polymerase chain reaction (qPCR) amplification (1,2) has been well established, with published guidelines for protocol optimization (3,4), interpretation of reaction kinetics, and accurate results reporting (5–9). Isothermal amplification is an alternative approach for nucleic acid amplification that does not require temperature cycling (10–12). Many isothermal amplification techniques allow rapid amplification reactions (13,14), do not require expensive equipment for thermocycling, allow both simple visual and fluorescence-based multiplex readouts (15–18), and have the potential to improve diagnostics in point-of-care and limited-resource settings (19). Nucleic acid quantification using real-time isothermal amplification has been described in many methods, including RPA (20), LAMP (18), NASBA (21), and RCA (22), by interpreting the standard dilution curves of exponential amplification profiles, an approach similar to the well-established one used in qPCR.

**Figure 1.**
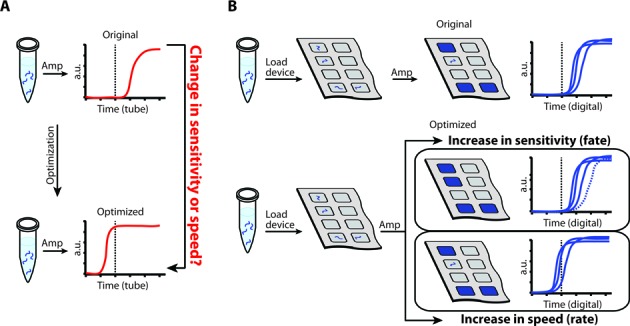
A schematic comparison of isothermal amplification in a tube (A), and in a digital single-molecule microfluidic device (B), responding to a change in conditions during optimization. (**A**) Increased non-digital reaction speed may either indicate a faster amplification of a few successful molecules and their products, or suggest that more template molecules initially participated in exponential amplification—indicating therefore an improved analytical sensitivity. Each template molecule is shown as a blue wavy line, and the accumulation of the amplification product is indicated as the solid red curves on the graphs. (**B**) The digital single-molecule method allows for independent measurements of reaction rate and analytical sensitivity during amplification of each template molecule (or lack thereof) within each well (squares in the gray device) to give rise to amplification product (blue-filled squares in the device and solid blue curves on the graphs).

Microfluidic methods have contributed to shorter amplification reaction times (23) and reduced reaction volume, and enable digital quantification as an alternative to real-time (kinetic) quantification (24–26). When the digital method is applied to PCR, absolute and reliable quantification can be achieved (27–30). Reliable quantification via digital methods has also been shown for some isothermal amplification reactions, such as RPA (31), RT-LAMP and LAMP (32,33), and RCA (34). In the digital amplification-on-a-chip format, a solution containing templates is loaded into a device with multiple wells at a low enough volume that each well is likely to contain either 0 or 1 template molecule. Every individual template that amplifies gives rise to a fluorescent signal in its separate well. The number of positive wells can then be counted optically to deduce starting concentration of the target nucleic acid. If all loaded template molecules amplify, absolute quantification is possible, but this only occurs in well-optimized amplification reactions (28). However, even if not all loaded template molecules amplify, digital quantification still provides precise comparisons of the relative template concentrations (35). The key parameter for evaluating the performance of an amplification reaction is its ‘digital efficiency,’ the percentage of templates that successfully amplify from the total template pool. Digital efficiency impacts assay accuracy (the ability to accurately quantify a loaded number of template molecules), and impacts analytical sensitivity (the ability to detect even a small number of template molecules in a reaction)—the standard parameters in the ‘Minimum Information for Publication of Quantitative Real-Time PCR Experiments’ (5). The limit of detection (LOD) of any analytical procedure considers the background signal (36), therefore high analytical sensitivity requires low false positive signal, together with the best performance. LOD is generally expressed as the analyte concentration corresponding to the sample blank value plus three standard deviations. High sensitivity is critical for the practical use of isothermal amplification chemistries. For example, to detect HIV viral load at 50 copies/ml of blood plasma (37,38) with no more than the 100–200 μl of plasma obtained from a fingerprick in limited resources settings, the method of detection should be sensitive enough to reliably detect 5–10 molecules of HIV RNA in a reaction. In this paper, we utilize the SlipChip digital platform (31,35,39), which allows both single-molecule amplification (39) and real-time monitoring of amplification reactions for each template molecule (40) (Figure 1). We selected a digital platform because it is robust and it has been well-characterized and validated (24–25,27–35,39), specifically on SlipChip devices with endpoint and real-time imaging (31,32,35,39,40).

During optimization, primer variants and reaction conditions must be compared empirically to ensure the nucleic acid sequences of interest are being detected reliably through amplification. While PCR approaches for selecting the best conditions are well-standardized (41), guidelines for optimizing isothermal amplification reactions are not as well developed. As a rule, in qPCR the best primer pair will yield the product with the lowest average quantification cycle, Cq (the PCR cycle at which a target is quantified) at equal template concentrations and under identical experimental conditions (41). In qPCR systems, the Cq value is dependent only on amplification efficiency, the number of starting template copies, and background fluorescence. The best primer pair also provides the highest possible analytical sensitivity in a multi-molecule format (e.g. in a PCR tube or well plate) and the highest possible digital efficiency in a digital format.

Reaction speed would seem to be an attractive criterion of efficiency when estimating isothermal amplification reaction performance (42) as well as when testing primers (43) and conditions (44) during optimization, as it is more convenient than performing dilutions and determining the LOD, the template concentration that can be detected with reasonable certainty, e.g. 95% confidence (5) for each condition. However, this approach, widely used in qPCR, had not been rigorously tested for isothermal reactions, and we predicted that it may not hold for isothermal reactions due to differences in the way isothermal reactions proceed. While isothermal amplification is ‘chained,’ similar to PCR, and exhibits exponential kinetics, there are no cycles defined by temperature. In qPCR, all the steps of amplification are time-synchronized and the number of cycles is counted rather than the absolute time of the reaction being measured. Time is allotted for each process (denaturation, annealing, elongation) in each cycle; if this time is sufficient for each process to complete, one will often not detect any minor differences in the efficiency of each process for amplification of different copies of the template molecule. In contrast, in isothermal reactions all biochemical events take place in parallel and the total time of the reaction is measured rather than the number of cycles. Thus, we hypothesized that changes in kinetics of any of the processes will more noticeably affect the time to threshold in isothermal reactions, compared to qPCR.

There are many factors that could theoretically affect speed and analytical sensitivity in isothermal amplification reactions. Consider these three examples. First, sometimes more than one enzyme is used simultaneously (e.g. in NASBA (21), strand displacement amplification (SDA) (45), helicase dependent amplification (HDA) (46), isothermal and chimeric primer-initiated amplification of nucleic acids (ICAN) (47) and all reverse transcription isothermal amplifications are performed in one step, such as in one step RT-LAMP (48,49)). Some of these co-occurring biochemical reactions could influence each other in isothermal reactions (similarly to how reverse transcriptase inhibits amplification in the non-isothermal reaction RT-PCR (50)). Second, sometimes numerous annealing events have to be coordinated to prevent them from competing with each other (e.g. in LAMP). Some annealing events influence the speed of isothermal reactions dramatically, e.g. the annealing of turn back primers (43,51), which is required for amplification, and the optional annealing of loop primers (13) and stem primers (52) in LAMP. Although the products of amplification from the extensions of two additional loop primers in LAMP do not contribute to the pool of exponentially amplifying DNA sequences nor are they required for the basic amplification mechanism (48), their presence is known to improve reaction sensitivity (13). Stem primers’ effect on amplification reaction sensitivity has not been addressed in publications. Third, the absence of multiple denaturation steps in isothermal amplification reactions suggests that reaction speed would be dependent on the template's innate secondary structures. Thus, an isothermal reaction's speed and analytical sensitivity are related in a more complex way than in PCR and cannot be predicted *a priori*.

We hypothesize that each component of an ongoing isothermal amplification reaction may potentially affect the reaction in one of the following ways: (i) it may limit the reaction's speed (the time it takes for an amplification reaction to produce a threshold level of signal), (ii) it may influence each single template molecule's ‘fate’ (whether it is amplified giving rise to a detectable signal, or lost from the amplification chain) thus affecting the analytical sensitivity or (iii) it may affect both the fate and the rate of amplification events.

When isothermal amplification reactions take place in a multi-molecule format (e.g. in a PCR tube or well plate) it is difficult to identify whether a change in time to threshold observed upon a change in primers or reaction conditions is the result of a larger fraction of templates amplifying (improved ‘fates’) or a change in reaction rate only (Figure 1A). In contrast to a multi-molecule format, digital experiments would separately measure the ‘fate’ (expressed as digital efficiency) of template molecules, allowing a more sensitive reaction design (Figure 1B). Real-time imaging of singe-molecule amplification in each well measures both the change in ‘fates’ and also change in amplification ‘rates’ (expressed as time to positive) as a result of a new primer or reaction condition. Here, we asked whether accelerating isothermal amplification reactions in a multi-molecule format in response to an introduced change in conditions always reflects improved analytical sensitivity and digital efficiency. This is an important question to answer in order to determine whether the standard qPCR approach using kinetics comparisons (speed of amplification) to find the best primers and conditions is also applicable to isothermal reactions. In this paper, we tracked amplification of single template molecules of HCV 5′ UTR RNA in real time under several different RT-LAMP conditions, and compared these observations to data on real-time multi-molecule amplification reaction speeds performed in a well plate.

## MATERIALS AND METHODS

### Chemicals and materials

All common reagents were purchased from commercial sources with the exception of RTx Bst 2.0 enzyme mixture (provided by New England Biolabs, NEB). Commercial reagents used were the same as described in Sun *et al*. (35), with the exception of SUPERase-In™ RNAase Inhibitor (Ambion by Life Technologies, Carlsbad, CA, USA) and Ultra-Pure distilled DNAses and RNAses free water (Invitrogen by Life Technologies, Carlsbad, CA, USA).

### RNA template

AcroMetrix® HCV-S panel RNA was extracted either with the QIAamp Viral RNA Mini Kit (QIAGEN Inc., Valencia, CA, USA) or with Maxwell® 16 Viral Total Nucleic Acid Purification Kit (Promega, Madison, WI, USA) according to the manufacturer's instructions. Nucleic acid extractions were immediately diluted using Ultra-Pure distilled DNAses and RNAses free water (Invitrogen), partitioned into about 100 separate 10 μl aliquots and stored at −80°C. Each 10 μl aliquot was further diluted and re-aliquoted to use as a template in RT-amplification. RNA fragment sequence was determined using RT-PCR reaction and Laragen Inc sequencing services. RNA concentrations were estimated through dRT-PCR as described below.

### Estimation of HCV RNA concentration using dRT-PCR amplification on the SlipChip device

HCV viral RNA was added to the RT-PCR mix, which contained the following: 20 μl of 2X SsoFast EvaGreen SuperMix, 1.0 μl of each PCR primer (10 μM), 2.0 μl of BSA solution (20 mg/ml), 1.0 μl of SuperScript® III Reverse Transcriptase, 1.0 μl of SUPERase-In™ RNAase Inhibitor, 13 μl of nuclease-free water, and 1 μl of template solution. RT-PCR primers used for HCV RNA template quantification were described previously (39,53). The amplifications were performed using SlipChip devices and a custom built real-time instrument, using the following protocol: an initial 15 min at 50°C was applied for reverse transcription, then 2 min at 95°C for enzyme activation, followed by 40 cycles of 1 min at 95°C, 30 s at 55°C and 45 s at 72°C. Images of all 1280 wells on each device were acquired for each cycle at 72°C. After the final cycle, a final elongation step was applied for 5 min at 72°C. This thermal cycling program was applied to all real-time digital experiments except for those done with end-point read-out on the PCR master cycler machine (Eppendorf) where 33 cycles were selected as a single end-point imaging cycle. End-point readout was done as described previously (39). At least six RT-PCR amplification reaction replicas on SlipChip devices were done to determine RNA concentration, and this concentration was used as a reference for all the future experiments.

### Real-time measurements and real-time digital measurements

To confirm that time to threshold values (Cq) in a digital format correlates with the Cq in a multi-molecule format, we used custom-built real-time instrument imaging and software that allowed us to observe the process of amplification in each well initially containing a single template molecule. SlipChip devices have been used most often to see only the end-point amplification in each well. Here, we used real-time imaging of the chip (Supplementary Figure S1A) (40) to track the amplification progress of each well in real time and record amplification curves (Supplementary Figure S1B), as described below. We used digital measurements of digital efficiency, real-time digital measurements of both digital efficiency and reaction rates, and then compared these results to real-time kinetic measurements performed in a well plate, done in parallel for each condition tested. Due to heterogeneity among the rates of amplification of different molecules (Supplementary Figure S2) the reported ‘time to positive’ in the digital experiments was selected as the time to a fluorescent signal in a first positive SlipChip device well (or the first few wells when they show a positive signal simultaneously), which is immediately followed by the appearance of a subsequent series of signals from other positive wells. During the selected times of the reactions, no false positive signals from the negative controls ever appeared for all the primers tests used.

### Fabrication and design of the SlipChip devices

SlipChip devices were fabricated, cleaned, assembled, and loaded as described previously (32,54). Each device contained a total of 1280 wells etched to a depth of 55 μm for a loading well volume of 3 nl on each side (6 nl when device is loaded from both sides); however, devices were always loaded from one side and the second half was filled with oil and used for a thermo-expansion volume (55).

### Real-time digital imaging

Real-time digital experiments were performed on a custom-built instrument that uses a Bio-Rad PTC-200 thermocycler with a custom machined block for thermal control/incubations at chosen temperatures. The block has a flat 3 in x 3 in area that accommodates microfluidic devices. The excitation light source was a Philips Luxeon S (LXS8-PW30) 1315 lumen LED module with a Semrock filter (FF02–475). Image Acquisition was performed with a VX-29MG camera, a Zeiss Macro Planar T F2–100mm lens, and a Semrock filter (FF01–540) for emission.

### Real-time digital analysis

Acquired images were analyzed using custom LabVIEW software. The data were analyzed by first creating a mask that defined the location of each reaction volume in the device. The masked spots were then used to extract the average intensity information of each digital well over the course of the experiment. Threshold was then manually set as half the height of the averaged and background-corrected maximum intensity, and the time to positive of each reaction was determined as the interpolated point at which the real-time curve crossed the defined threshold. Poisson statistics were used in automated software calculations of the loaded template concentrations, based on the percentage of wells that showed template presence.

### RT-LAMP primer design, and primer sets used

For primer design we used Oligo 7.0 software (Molecular Biology Insights, Inc., Cascade, CO, USA); alignment of available HCV 5′ UTR sequences was done using Geneious 6.1.6 software (Biomatters Ltd, Auckland, NZ) to select the most conserved fragments to position the 5′ and 3′ ends of FIP and BIP. HCV 5′ UTR fragment of interest secondary structure has been evaluated at different temperatures using NuPack, as RNA and as both single strands DNA form (56).

We followed recommendations on LAMP primer design from the Guide to LAMP Primer Designing on the EIKEN web site (57) and used the primers in recommended relative concentrations ranges (48). A few primer variants were experimentally tested via digital efficiency evaluation, and compared with HCV ‘best published primers’ (BPP) from the literature (58). We designed back primers (BIP, loopB and B3) ourselves and we modified the BPP set forward primers (FIP, loopF and F3) to place the important primer parts into the most conservative HCV sequence alignment fragments. We tested all planned primer alterations for forward and back primers independently, introduced them one by one, and measured the relative change in digital efficiency for at least three replicates, and used the BPP set performance in dRT-LAMP as a reference. We selected the primers with the highest digital efficiencies in dRT-LAMP amplification reactions and we named this set ‘digitally optimized primers’ (DOP):

- BIP 5′-TTGGGCGTGCCCCCGCAAGTTTTCAGTACCACAAGGCCTTTCGCGACC-3′
- FIP 5′-TCCAAGAAAGGACCCGGTCTTTTTCTGCGGAACCGGTGAGTAC-3′
- LoopB 5′-CTGCTAGCCGAGTAGTGTTG-3′
- LoopF 5′-GTCCTGGCAATTCCGGT-3′
- F3 5′-CCTCCCGGGAGAGCCATAG-3′
- B3 5′-GCACTCGCAAGCACCCTATC-3′

The same DOP set, modified to determine four circulating HCV genotypes by incorporation inosine bases, was used previously (40).

Four variants of DOP set were designed for testing the correlation of the speed and sensitivity of isothermal amplification. Through elongation of the F1c part of FIP, we designed ‘long FIP’ (LFIP) primer 5′-GGTTGATCCAAGAAAGGACCCGGTTTTTCTGCGGAACCGGTGAGTAC-3′ to use in a model DOP-LFIP primer set. Through elongation of the B1c part of BIP primer we designed ‘long BIP’ (LBIP) primer 5′-GAGATTTGGGCGTGCCCCCGCAAGTTTTCAGTACCACAAGGCCTTTCGCGACC-3′ to use in a model DOP-LBIP set. The variants of the DOP sets ‘no loop F’ (DOP-NLF) and ‘no loops’ (DOP-NL) were used to test the effect of loop primer presence on the speed and sensitivity of amplification. The DOP-NLF set was the same as the DOP but the loopF primer was excluded (only loopB primer was present). The DOP-NL set was the same as the DOP set but lacked both loopB and loopF primers.

### RT LAMP primers and conditions

Digital RT-LAMP using SlipChip devices, and RT-LAMP in well plates were performed with all the primer set variants in one step as described previously (40), with the following modifications: We used 1.7 μl of enzyme in 40 μl of total reaction mixture instead of 2 μl, and standard 3 μl of Acrometrix HCV-s RNA template solution (or nuclease-free water for negative controls). In all the primer sets, both the B3 and B2 parts of BIP served as gene-specific primers for reverse transcription. Enzymes tested were: (i) EM (EIKEN), used for all the experiments, and (ii) a mix of Bst 2.0 (NEB) with an experimental lot of RNaseH active thermostable reverse transcriptase Rtx (NEB) used only for one experiment to compare different enzymes (Figure 5). Amplification products detection was performed with calcein (FD) (EIKEN). Real-time bulk RT-LAMP data analysis was done as described previously (40).

## RESULTS

To test whether reaction rate and digital efficiency always correlate in the case of isothermal reactions, we used digital amplification on SlipChip to reanalyze two common approaches believed to improve performance of LAMP: (i) selecting primers in the recommended melting temperature (*T*_m_) ranges to ensure correct annealing order (48), and (ii) using loop primers to increase speed and sensitivity (13). First, we used the digital optimization process described in this paper to optimize a set of primers, which we call ‘digitally optimized primers’ (DOP). In the comparisons of speed and efficiency, we compared this DOP set to four other primer set variants: DOP with an elongated BIP primer (DOP-LBIP), DOP with an elongated FIP primer (DOP-LFIP), DOP with no loopF primer (DOP-NLF), and DOP with no loops (DOP-NL). We also tested for a correlation between digital efficiencies and rates of reactions performed with each of two variants of reverse-transcriptase/Bst polymerases enzymes mixtures.

### The effect of turn back primers (FIP and BIP) on amplification speed and sensitivity

**Figure 2.**
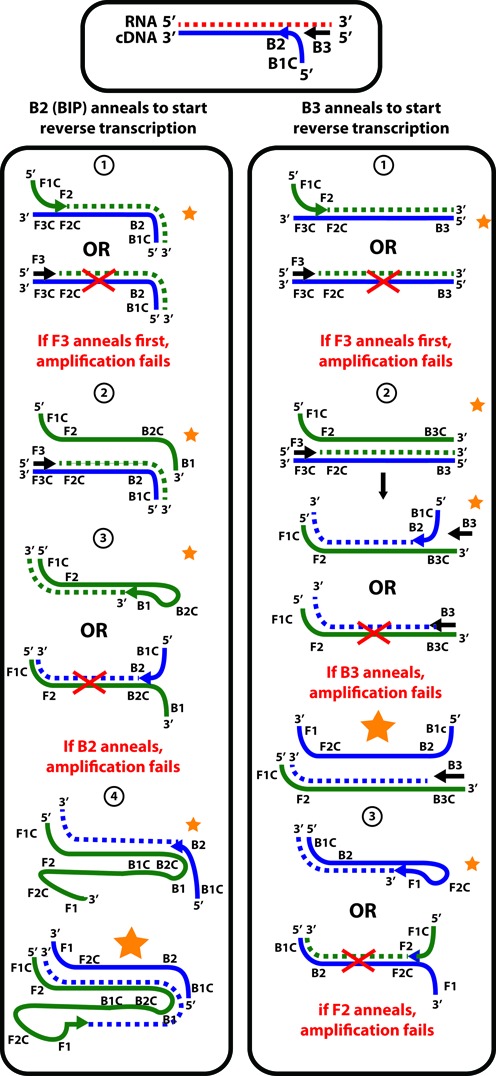
Diagram of annealing events during RT-LAMP amplification. Synthesis of cDNA starts either from the B3 primer or from the BIP primer (which consists of B2 and B1c fragments). The RNA template is degraded through RNAseH activity of reverse transcriptase. Afterward, competition between different primers’ annealing occurs, affecting the ‘fate’ of each cDNA molecule and its products (i.e. whether they will remain in or be excluded from the pool of amplifying molecules). F1 and B1 annealing should occur before the F2 and B2 annealing, and B2 and F2 annealing should occur before B3 and F3 annealing for optimal amplification.

We compared the reaction rates and digital efficiencies of reactions using DOP and those using either the DOP-LBIP primer set or the DOP-LFIP primer set. The rationale for this experiment is that we assumed that the order of the primer annealing in a LAMP reaction strongly affects digital efficiency (Figure 2). Ideally, F1c and B1c anneal first, F2 and B2 anneal second, and F3 and B3 anneal last. These considerations are in line with the recommendations listed in “A Guide to LAMP Primer Designing" (57).

Primer annealing to template depends on each primer's *T*_m_ and the template's secondary structure. The interplay of these two factors is addressed through the concept of net *T*_m_, which is the temperature at which half of the template is bound by the oligonucleotide (59,60). While we do not know the exact secondary structure of a LAMP amplicon under reaction conditions, we modeled the predicted secondary structure for our amplifying DNA fragment using NuPack software (56). This modeled secondary structure appears to be very similar to the published secondary structure for HCV 5′ UTR RNA (61,62) (Supplementary Figure S2). It's possible that making B1c and F1c longer in LBIP and LFIP not only increased the primers’ *T*_m_ (Table 1), but also affected their net *T*_m_ as a result of positioning the B1c and F1c ends into template regions that were richer in secondary structures (Supplementary Figure S2). Making B1c and F1c longer may have affected the probability of non-paired state at the 5′-end regions of the turn back primers, which is known to influence amplification (43). If secondary structures are significantly more abundant in the template fragments, only empirical testing can verify that primers work better after optimization via increased calculated *T*_m_.

**Table 1.** Annealing sequences of the standard primer set and the elongated primer variants, the nucleotide sequence, and the predicted melting temperatures (*T*_m_) at a standardized concentration of primers

Annealing sequence	Nucleotide sequence (5′ to 3′)	*T*_m_
F1c of FIP (as in DOP)	TCC AAG AAA GGA CCC GGT C	68.4ºC
F1c elongated (of FIP) to use in DOP-LFIP	GGT TGA TCC AAG AAA GGA CCC GG	70.7ºC
F2 (of FIP)	T CTG CGG AAC CGG TGA GTA C	70.2ºC
F3	CCT CCC GGG AGA GCC ATA G	65.9ºC
B1c of BIP (as in DOP)	TTGGGCGTGCCCCCGCAAG	73.7ºC
B1c elongated (of BIP) to use in DOP-LBIP	GAGATTTGGGCGTGCCCCCGCAAG	76.3ºC
B2 (of BIP)	CAGTACCACAAGGCCTTTCGCGACC	73.7ºC
B3	GCACTCGCAAGCACCCTATC	66.0ºC

**Figure 3.**
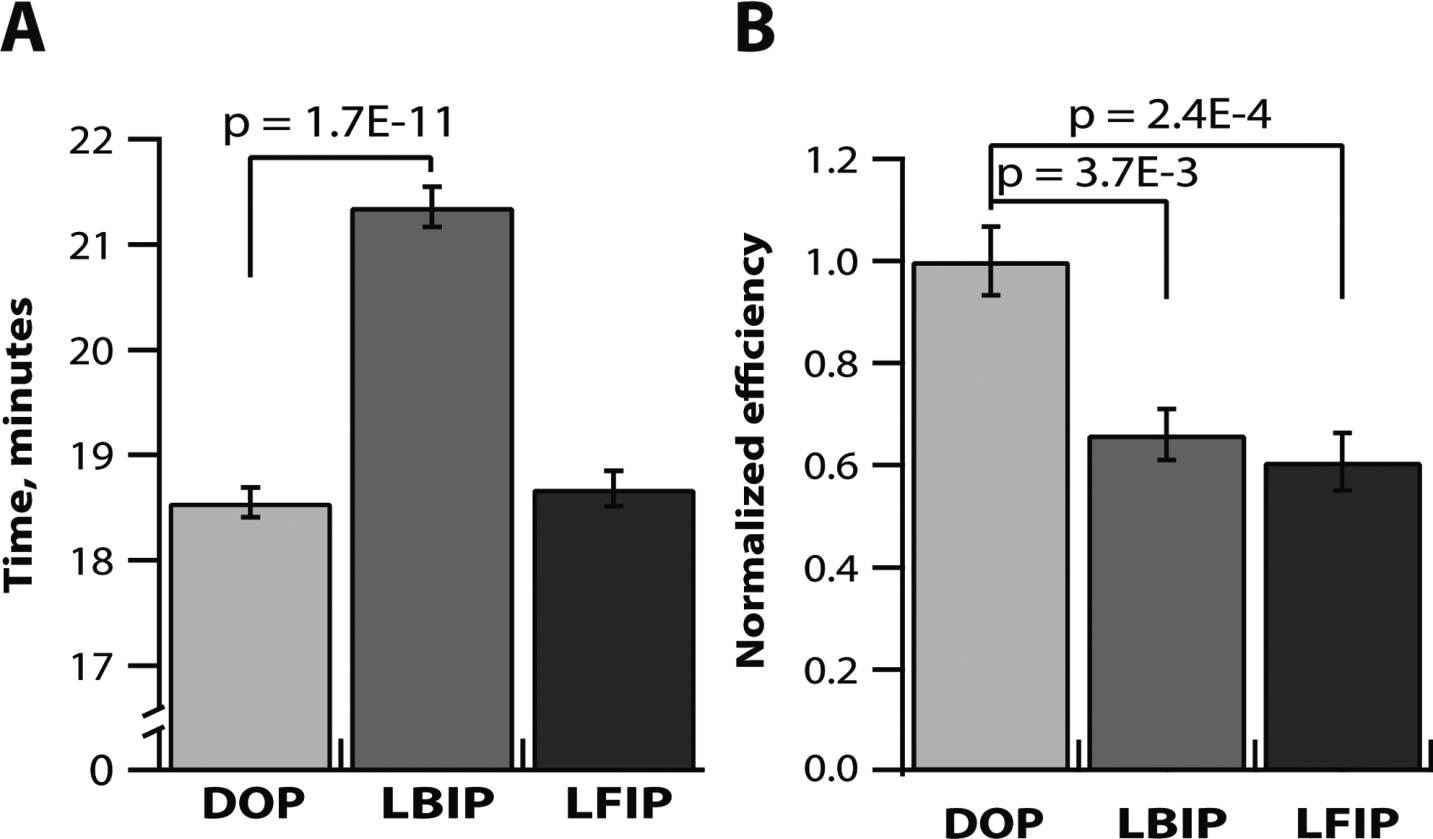
Comparison of time to positive and RT-LAMP reaction efficiencies for the ‘digitally optimized primers’ (DOP) set and the elongated BIP and FIP sets (DOP-LBIP and DOP-LFIP) through real-time measurements of reaction speeds (time to threshold values in min) and real-time digital measurements of the template molecules’ ‘fates’ (expressed as normalized digital efficiencies). (**A**) Plot comparing times to positive in multi-molecule experiments for standard and elongated BIP and FIP primers with 5′ ends placed into the secondary structures. *N* = 9–24; (**B**) Plot of normalized relative digital efficiencies for single-molecule experiments using standard and elongated BIP and FIP primers. *N* = 3–8. *P*-values are above brackets and error bars designate S.E.

In our experiments, for both DOP-LFIP and DOP-LBIP primer sets, elongation of FIP or BIP primers led to a drop in digital efficiency compared to the DOP set. For DOP-LFIP, the digital efficiency dropped by 40% ± 6% (S.E.) of DOP (*P* = 2.4 × 10^−4^) and for DOP-LBIP the digital efficiency dropped by 34% ± 5% (*P* = 3.7 × 10^−3^) (Figure 3B). However, the change in reaction speed was different for the two primer sets. In the case of elongated BIP, the drop in efficiency was accompanied by a decrease in speed (longer time to positive), as would be expected in analogy to qPCR. Here, time to positive increased from 18.6 ± 0.1 min to 21.4 ± 0.2 min (*P* = 1.7 × 10^−11^). Surprisingly, in the amplification reaction using elongated FIP, the drop in efficiency was not accompanied by a change in time to positive; the DOP-LFIP time to positive was similar to DOP, 18.7 ± 0.2 min (*P* = 0.585) (Figure 3A). Thus, reaction speed and digital efficiency do not always correlate in isothermal amplification reactions.

These results suggest that selecting primers for LAMP in the recommended *T*_m_ ranges isn't enough to ensure better reaction performance, as in one case we observed a drop in reaction speed and in both cases we observed a decreased ability to determine template concentrations with high sensitivity (detected as a drop in digital efficiency). Measurements of reaction speed alone did not allow a reliable comparison of the tested primers, whereas digital measurements of the cumulative fates of the template molecules (Figure 3B, Supplementary Figure S3A) provided a tool for a direct comparison of primers efficiencies. These experiments specifically show that, in some cases, two isothermal reactions performing at the same speed may differ in their digital efficiencies.

### The effect of loop primer presence on amplification speed and sensitivity

**Figure 4.**
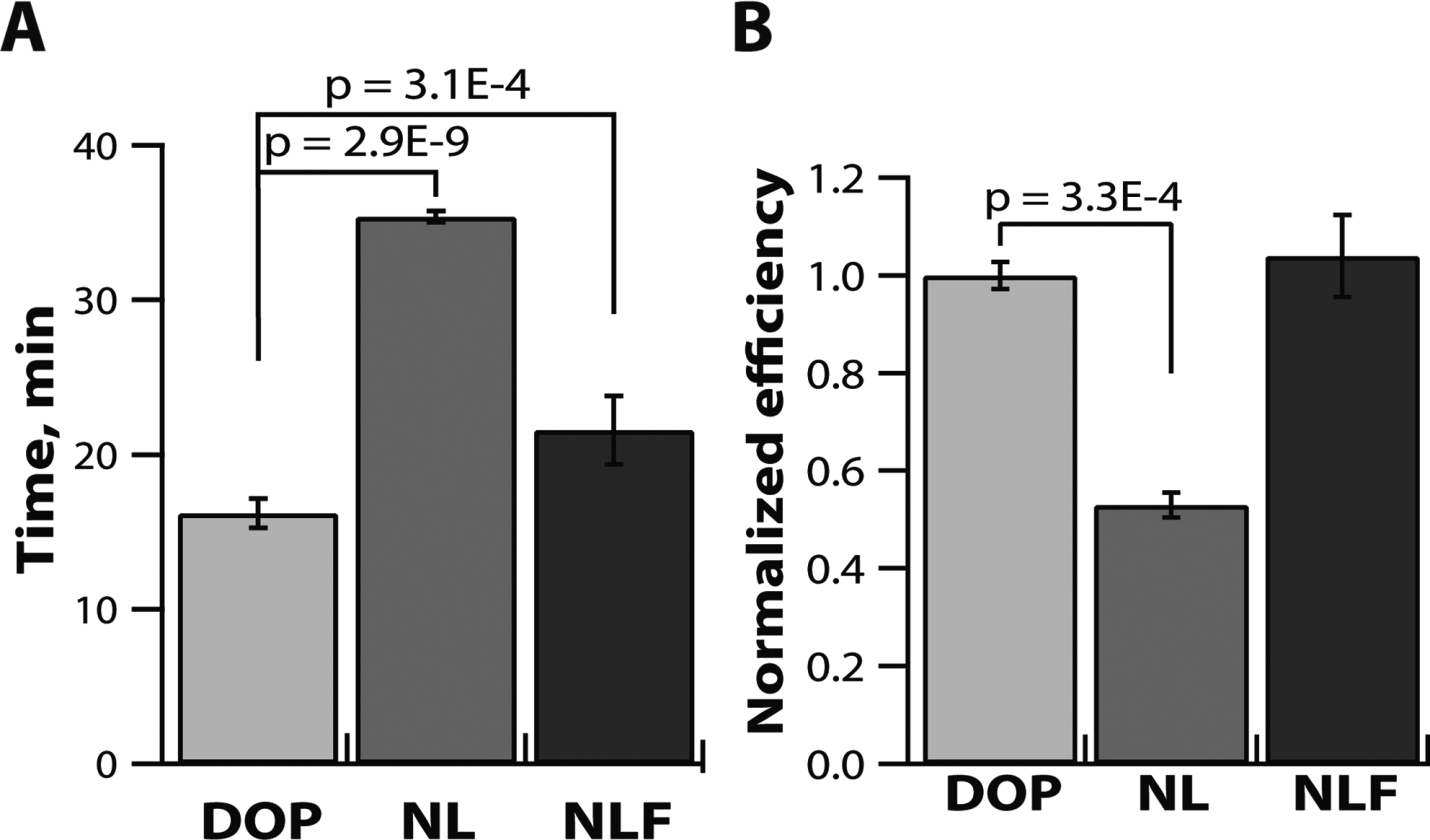
Real-time measurements of reaction speeds (time to threshold values in min) and real-time measurements of single molecule amplification fates (expressed as normalized digital efficiencies). (**A**) Plot comparing times to positive in a well plate for the ‘digitally optimized primers’ set and the DOP set with no loop primers (DOP-NL) and the set with no loop F primer (DOP-NLF), *N* = 12–24. (**B**) Normalized relative digital efficiencies with DOP and DOP-NL and DOP-NLF. *N* = 4–8. Significant *P*-values are designated above the brackets; error bars are S.E.

We compared the effect of loop primers on digital amplification efficiency, as well as on the speed of the reaction in both digital and multi-molecule formats, using the DOP set and its two variants: the DOP set with no loops (DOP-NL) and the DOP set with no loop F (DOP-NLF). The DOP-NL set was significantly slower and about half as efficient 53% ± 2% (S.E.) as the DOP set. The average times to positive in the reaction with DOP were 18.5 ± 0.1 min, and those with DOP-NL were 37.4 ± 0.7 min (Figure 4A and B). This result supports the published observation (13) that using loop primers improves primarily the speed of the reaction and also its sensitivity.

When we compared the primer set with no loop F (DOP-NLF) to the DOP set, which contained both loops, we found that the speed of the reaction with DOP-NLF (23.2 ± 0.2 min) was about 4.7 min slower than the DOP set (18.5 ± 0.1 min; *P* = 3.1 × 10^−4^) (Figure 4A). However, surprisingly, the digital efficiencies did not differ significantly between the DOP and DOP-NLF sets (*P* = 0.37). This comparison of the corresponding reaction efficiencies showed that the presence of only one loopB primer was sufficient to maintain the same ability to determine template concentrations with high sensitivity (detected as digital efficiency), as with both loop primers, despite the drop in reaction speed (Figure 4B).

The lack of correlation between reaction speed and efficiency in the case of only loopB primer presence in the reaction mixture may be partially explained by the fact that the products of loop primer amplification by design (13) cannot efficiently participate in subsequent exponential amplification. Despite having a primary ‘signal amplifying’ function, loop primers still improve digital efficiency (Figure 4B), which is in agreement with previous work showing their positive effect on sensitivity (13). For the first time we show that despite a drop in reaction speed, having just one loop primer in a reaction mixture is sufficient to maintain the high digital efficiency seen in experiments containing both loop primers. Measurements of speed alone did not allow reliable detection of the changes in sensitivity that resulted from different loop primers being present, whereas digital measurements of the cumulative fates of the template molecules (Figure 4B, Supplementary Figure S3B) provided a tool for a direct detection of the changes. These experiments specifically show that, in some cases, two isothermal reactions performing at the same digital efficiency may differ in their reaction speeds.

### The effect of using different enzyme mixtures on amplification speed and sensitivity

We also tested whether different enzyme mixtures affected reaction speeds and digital efficiencies in a correlating way. Reverse transcription adds a few uncertainties to subsequent amplification outcomes. First, its efficiency directly affects the fate of RNA molecules—whether they are reverse transcribed and used as cDNA copies in a subsequent amplification chain, or lost from the template pool. Second, the temperature at which different enzymes exhibit optimal activity affects the outcome of reverse transcription of the secondary structure-rich templates (63), especially when gene-specific primers are used, or when reaction is done as a one-step RT-LAMP performed at 63°C. Third, reverse transcriptase may interfere with polymerase performance (50), as reverse transcriptase binds to the primers/DNA complexes and may also exhibit some limited DNA/DNA polymerase activity.

**Figure 5.**
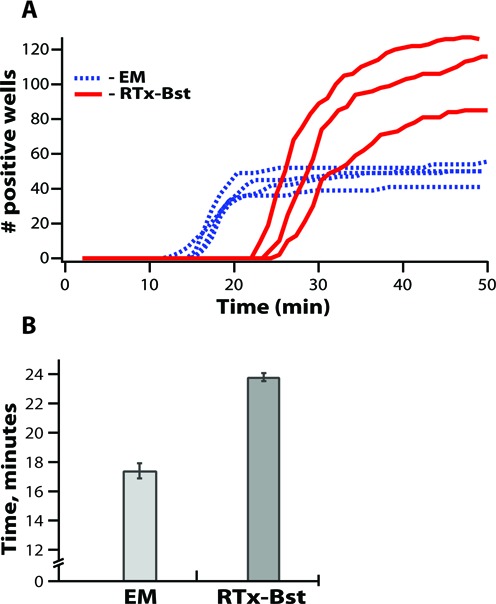
Comparison of the effect of two different RT-LAMP enzyme mixtures on amplification using (A) real-time, digital single-molecule and (B) real-time bulk approaches. (**A**) Real-time digital measurements of single-molecule amplification fates and rates in a microfluidic device shown as the number of wells that reached a signal threshold over time in each experiment (*N* = 3). We compared a commercially available enzyme mixture (EM, blue dashed lines) and an experimental lot of RTx Bst 2.0 enzyme mixture (red solid lines). (**B**) Real-time measurements of reaction speeds (time to threshold in min) in a multi-molecule format. In all enzyme experiments we used DOP primers with an FIP primer identical to the one from the BPP set. Error bars are S.E. and *N* = 3.

We used digital amplification to test an enzyme mixture of RNAseH active thermostable reverse transcriptase RTx and Bst 2.0 polymerase enzymes (from NEB). Our preliminary check of performance of this enzyme mixture showed later times to positive compared to an analogous reaction using a commercial enzyme mixture (EM, from EIKEN). However, when we tested how many HCV RNA templates were correctly detected from a known number of loaded RNA templates, we discovered that despite being slower than EM, the RTx Bst 2.0 enzyme mixture provided higher digital efficiency (Figure 5). These data show once again that isothermal reaction speed and efficiency do not always correlate, and could be untangled using digital measurements of the cumulative fates and rates of the template molecules (Figure 5A, Supplementary Figure S3C), but not using multi-molecule format alone.

### Characterizing ‘digitally optimized primers’ (DOP) and ‘best published primers’ (BPP) using digital experiments and experiments in a multi-molecule format

**Figure 6.**
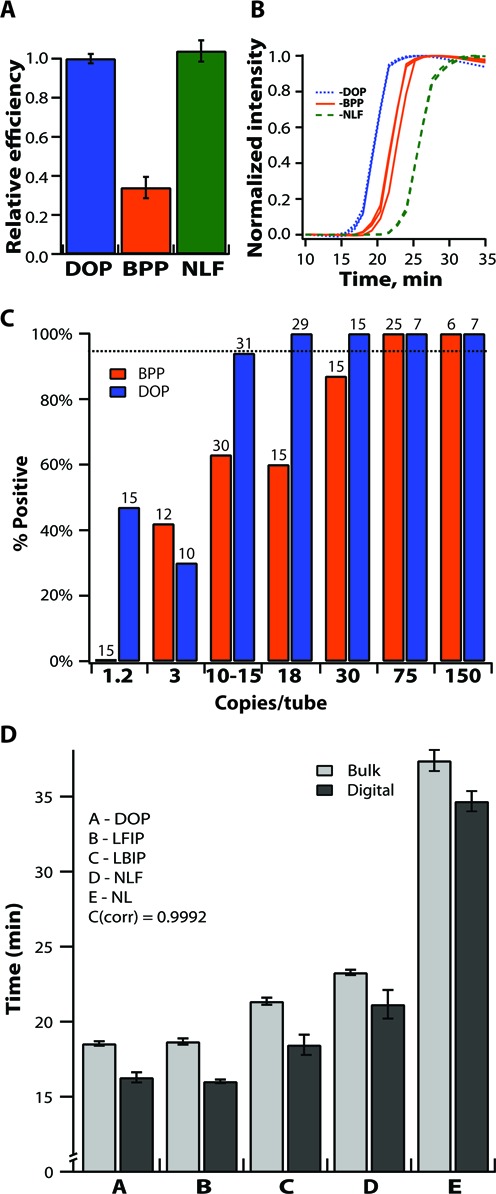
(**A**) The digital efficiency of the ‘best published primers’ (BPP) and the no loop F (DOP-NLF) sets normalized to the ‘digitally optimized primers’ (DOP) set, *N* = 6. (**B**) Normalized fluorescence intensity over time for amplification using the BPP (orange lines), DOP (blue lines) and DOP-NLF (green dashed lines) sets, *N* = 3. (**C**) Percent of positive wells in a PCR well plate at different template concentrations with the DOP and BPP sets (N indicated above each bar). (**D**) Measured times to positive of LAMP reactions in a multi-molecule format in PCR well plates (light gray) and in the digital format on a microfluidic device (dark gray) for all primer sets. Error bars indicate S.E., *N* = 4–8 in digital; *N* = 16–40 for well plates.

To address whether digital efficiency correlates with analytical sensitivity (measured using a multi-molecule format), we compared the DOP set to a set of primers taken from the literature for HCV 5′ UTR RNA, which we refer to as ‘best published primers’ (BPP) (58). We used digital measurements of efficiency, and real-time digital measurements of both efficiency and reaction speed. We also did real-time kinetic measurements of reaction speed and determined analytical sensitivity (determined as the limit of detection, LOD) in a multi-molecule format using standard PCR well plates for each condition. We found good agreement between digital efficiency and LOD measured in well plates for both the DOP and BPP sets. The normalized BPP digital efficiency measured in a microfluidic device was 34% of the DOP digital efficiency (Figure 6A) (*P* = 1.05 × 10^−6^). In the multi-molecule format, the LOD for the BPP set was determined to be 75 template copies/10 μl, while the LOD for DOP set was found to be 18 copies/10 μl (Figure 6C). The LOD values in PCR well plates and the digital efficiencies measured in microfluidic devices correlated well in this example. At a very low template concentration of ∼1.2 copy/10 μl, the DOP set enabled detection of 44% of the wells in the well plates; whereas there were no positive signals detected at the same template concentration with the BPP set (Figure 6C), which is a result of the higher analytical sensitivity of the DOP set.

To ensure that in all cases the results of the reactions in digital format were in accordance with those of reactions performed in a multi-molecule format, we measured the absolute time of the reaction in both formats (Figure 6D). The single-molecule enzymatic reaction start time was stochastic. Due to heterogeneity among the rates of amplification of different template molecules (Supplementary Figure S3), the reported ‘time to positive’ in the digital experiments was defined as the time to the first positive well (fluorescent signal) in a microfluidic device that was immediately followed by a subsequent series of signals from other positive wells. We found that for all primers used in our experiments under similar conditions, the times to positive in a digital format in a microfluidic device correlated well with the times to positive in multi-molecule reactions performed in a PCR well plate (Figure 6D). Our data indicate that the digital format resulted in a faster readout (Figure 6D), which is consistent with the higher concentration of template molecules in the digital reactions. While we started with identical solutions for multi-molecule and digital experiments, the effective concentration of single template molecules confined in microfluidic wells on a digital microfluidic device was ∼5 times higher than the concentration of the templates in corresponding reactions performed in a PCR well plate, because ∼ 80% of wells on the digital device lacked template molecules and therefore all of the template molecules were concentrated into ∼20% of the wells. This correlation (Figure 6D) between the times to positive of multi-molecule reactions and earliest amplification reactions in corresponding digital experiments (40) is consistent with the ‘winner takes all’ dynamics in multi-molecule amplification: the products of the first few successful amplification events become the primary source of amplicons for subsequent exponential reactions. We also plotted the average times to threshold values for BPP, DOP and DOP-NLF sets (Figure 6B) to illustrate that the relative efficiency of a primer set cannot be deduced through reaction speed alone. Time to positive signal was shorter for DOP (18.5 ± 0.1 min) compared to BPP (21.9 ± 0.2 min) sets, but the DOP NLF set had a longer time to positive (23.2 ± 0.2 min), although the DOP NLF set had the same efficiency as the DOP set (Figure 4B).

Digitally optimized primers had better analytical sensitivity compared to the best published primers set. The LOD for the DOP set was 18 molecules in 10 μl, while the LOD for the BPP set was 75 template molecules in 10 μl. However, in our multi-molecule format experiments, before we could determine these LOD values with statistical significance we had to test 99 PCR wells for the DOP set and 103 PCR well plate wells for the BPP set. In contrast, in the digital experiments comparing DOP and BPP sets, just one device per condition was enough to observe clear differences in the sensitivity of detection of loaded templates, and additional replicates confirmed statistical significance.

## DISCUSSION

Isothermal reactions provide a useful tool for nucleic acid amplification tests, particularly in point-of-care settings. Designing reliable tests requires finding the best isothermal amplification primer variants and reaction conditions. The digital format provides an invaluable tool for assessing the efficiency of an isothermal amplification reaction by directly detecting the percentage of successfully amplified template molecules from the known number of loaded template molecules. Our results show that digital efficiency correlates with analytical sensitivity, and that amplification reaction speed in a digital format correlates with reaction speed in a multi-molecule format (e.g. in a PCR well plate or tube). Thus, observations made about digital efficiency and reaction speed in nanoliter-scale volumes are directly applicable to the same reactions performed in a large-volume, multi-molecule format.

Applying the digital method to isothermal amplification experiments revealed a number of surprising results that contradict the intuition derived from qPCR experiments. First, and perhaps most interestingly, reaction speed does not correlate with digital efficiency (and analytical sensitivity) in isothermal amplification reactions. Specifically, testing FIP and BIP primer variants showed that the digital efficiency in one-step RT-LAMP reactions may be significantly higher for one of the tested primer variants, even without an observed change in the speed of the reaction (Figure 3). We also found a lack of correlation between speed and sensitivity (digital efficiency) in the experiments using different enzymes mixtures, where we observed reactions with higher digital efficiency having substantially longer times to positive (Figure 5).

Digital experiments confirmed that the presence of two loop primers in the LAMP reaction mixture slightly improved sensitivity to determine template concentration, in addition to their primary function of accelerating the accumulation of amplification products (13). However, an unexpected result was that having just one loop primer in a reaction mixture was sufficient to maintain the same improved digital efficiency, despite the expected partial drop in reaction speed compared to reactions containing both loops (Figure 4).

We conclude that the well-known qPCR approach for selecting optimal primers and conditions based on earlier times to positive is not applicable to all isothermal amplification reactions. In all of the reaction conditions we tested, deriving conclusions about optimization based only on observed changes in reaction speed could have been misleading. As a consequence, a kinetic-based evaluation of an isothermal reaction's performance (e.g. an evaluation based on the proposed isothermal doubling time (IDT) parameter (42)), would not discriminate between a slow, sensitive reaction, and a less sensitive (e.g. inhibited) reaction. Therefore, faster reaction speed is not an appropriate way to determine better reaction conditions or primers in the case of isothermal amplification reactions. Detailed analyses of optimization processes are typically not reported for new assays. The final analytical sensitivities of newly developed isothermal assays are either reported through LOD (64) or more typically evaluated by using 10-fold serial template dilutions that are then compared to the sensitivities of a standard PCR method as a way to demonstrate the value of each developed isothermal test (65–67).

An alternative approach to accurately evaluate different primer variants or conditions used in isothermal reactions is to perform experiments to estimate a limit of detection (LOD) (5) in a multi-molecule format for each introduced change in reaction conditions. However, this approach has a number of disadvantages: (i) sometimes a single introduced change in conditions may only slightly affect analytical sensitivity (ii) some introduced changes may have cumulative or interactive effects on analytical sensitivity, (iii) LOD experiments are not easy to perform at low dilutions especially for RNA due to its potential degradation, (iv) experiments must be done side-by-side for both tested conditions to exclude variation related to reagent freshness and reaction setups, and (v) a large number of replicates is required to establish statistical power.

Using digital methods during optimization can be a reliable tool for finding primers and conditions that allow the best analytical sensitivity in a standard multi-molecule format—providing faster results and requiring lower replication. To further illustrate the advantage of digital measurements in optimization, we performed a back-of-envelope analysis of a question: How many experimental replicates are needed (i.e. what is the ‘sample size’ necessary) to distinguish a change in digital efficiency (Target Difference, *TD*) between two reaction conditions in a digital format? We sought to answer this question in a way that would be applicable to both single-molecule amplification (e.g. digital formats) and multi-molecule amplification (e.g. in a PCR tube or well plate). In both cases, we calculated the minimum number of replicates (*N*) required to differentiate with statistical power which reaction had higher digital efficiency.

First, in the context of digital experiments, we calculated the standard deviation for the number of positive wells in a single device (39,68,69). For a device with 1280 wells of 3 nl and a concentration of 5 × 10^4^ molecules/ml, the standard deviation σ of ln(λ) is 0.075 (Equation 1):

(1) \begin{equation*} \sigma = \frac{1}{{\lambda \nu \sqrt {\frac{n}{{e^{\nu \lambda } - 1}}} }} \end{equation*}

Here, λ is the concentration in molecules/ml, ν is the well volume in ml, and *n* is the total number of wells in the digital device. We calculated TD as an absolute difference between the natural logarithms of two measured efficiencies (for this example a 20% difference in efficiency was selected). Next, we calculated the standardized difference, SD = 2.98, from:

(2) \begin{equation*} {\rm SD} = \frac{{{\rm TD}}}{\sigma } \end{equation*}

Finally, we calculated the minimum number of experimental replicates (*N*) required to achieve the TD (70):

(3) \begin{equation*} N = \frac{2}{{{\rm SD}^2 }} \times C_{{\rm p,power}} \end{equation*}

Here, C_p,power_, a constant defined by the combination of *P*-value (typically set to 0.05) and statistical power (set to 95%), is equal to 13.0 (70). Under these assumptions, *N* ∼ 3 (2.93), or only three SlipChip devices for each of the two conditions being compared are necessary to establish a 20% difference in detection efficiency between these reactions with 95% confidence and a *P*-value of 0.05. To establish a 25% difference in efficiency with the same parameters, we would need only N ∼2 (1.76) SlipChip devices.

Next, using the same approach, we calculated the theoretical number of replicates needed to achieve this level of statistical power in a standard, multi-molecule reaction when using ∼ 1 template copy per reaction (Equations 1-3). If one uses 10 wells (of 10 μl each in a well plate) per experiment, each loaded with 1 template molecule/well, 90 independent trials would be necessary, for a total of 900 reactions per condition (or 9 trials using 100 tubes each) which is impractical. Pragmatically, experiments are not done on this scale and therefore it has not been possible to optimize reactions by directly measuring small differences in detection efficiency, whereas digital experiments open this possibility.

The digital format provides accurate measurements of reaction efficiency, independent of reaction speed and we suggest that it provides an efficient tool for optimizing new assays based on isothermal amplification reactions. Isothermal amplifications chemistries beyond RT-LAMP should also be tested for the lack of correlation between reaction speeds and analytical sensitivities. We anticipate that digital methods will be useful both to understand mechanistic details of various isothermal amplification reactions and to improve these reactions for practical applications.

## Supplementary Material

SUPPLEMENTARY DATA
