# Supplementary material for: Lack of correlation between reaction speed and analytical sensitivity in isothermal amplification reveals the value of digital methods for optimization: validation using digital real-time RT-LAMP
Source: Nucleic Acids Res. 2015 Sep 10;44(2):e10. doi: 10.1093/nar/gkv877 (PMC4737171; doi:10.1093/nar/gkv877)
Supplement: SUPPLEMENTARY DATA [file supp_44_2_e10__index.html]

Lack of correlation between reaction speed and analytical sensitivity in isothermal amplification reveals the value of digital methods for optimization: validation using digital real-time RT-LAMP — SUPPLEMENTARY DATA 

# Lack of correlation between reaction speed and analytical sensitivity in isothermal amplification reveals the value of digital methods for optimization: validation using digital real-time RT-LAMP

## SUPPLEMENTARY DATA

- SUPPLEMENTARY DATA
