# Supplementary material for: Lack of correlation between reaction speed and analytical sensitivity in isothermal amplification reveals the value of digital methods for optimization: validation using digital real-time RT-LAMP
Source: Nucleic Acids Res. 2015 Sep 10;44(2):e10. doi: 10.1093/nar/gkv877 (PMC4737171; doi:10.1093/nar/gkv877)
Supplement: SUPPLEMENTARY DATA [file supp_gkv877_Isothermal_NAR_SI_revised_9.1.15.pdf]

Lack of correlation between reaction speed and analytical sensitivity in isothermal amplification reveals the value of digital methods for optimization: validation using digital real-time RT-LAMP

Eugenia M. Khorosheva, Mikhail A. Karymov, David A. Selck and Rustem F. Ismagilov*

Division of Chemistry and Chemical Engineering, California Institute of Technology, 1200 East California Blvd., Pasadena, California, 91125, United States

* To whom correspondence should be addressed. Tel: (626)395-2333; Fax: (626) 568-8743; Email: rustem.admin@caltech.edu

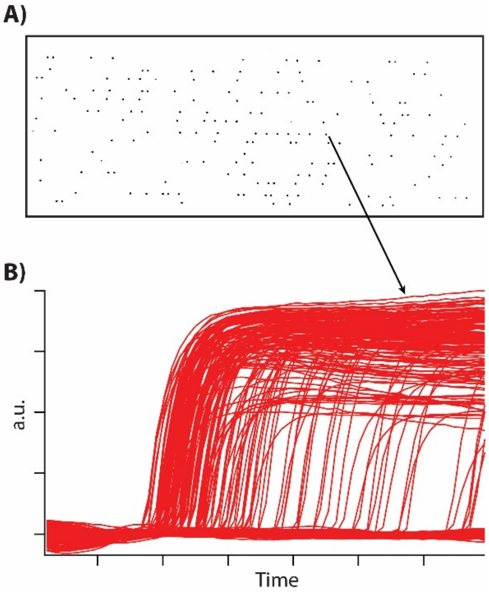

Figure S1. Real time imaging of single template amplification reactions using a 1,280-well SlipChip device. (A) A schematic of the final digital readout at the end point and (B) a plot of single template molecule amplifications in each well, tracked in real time. The arrow indicates the change in intensity (a.u.) over time of a single well due to the amplification process (B) and the same well's location in the device shown in (A).

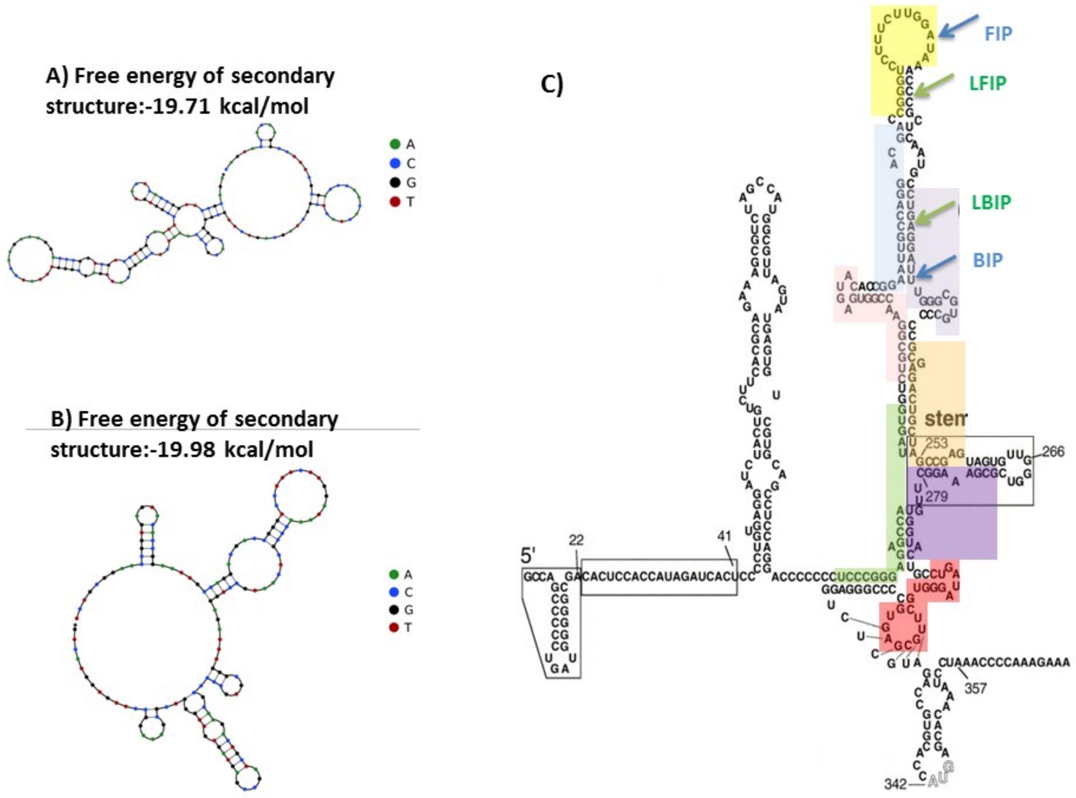

Figure S2. HCV LAMP amplicon DNA secondary structures (minimum free energy structures at 37.0°C), for cDNA analogous strand (A) and for complementary strand (B) as modelled using NuPack (1). (C) HCV 5'UTR RNA secondary structure, as published (61,62). Blue arrows mark the positions for F1 and B1 ends of BIP and FIP primers from DOP set. Green arrow mark the positions for F1 elongated and B1 elongated ends of LFIP and LBIP primers from DOP–LFIP and DOP-LBIP sets respectively. Color-marked fragments of nucleotide sequence stand for BPP set primers annealing sequences for reference (greenF3, pink – loopF, blue-F2, yellow – F1c, light purple - B1c, warm yellow – loopB , dark purple – B2 and red – B3).

A)

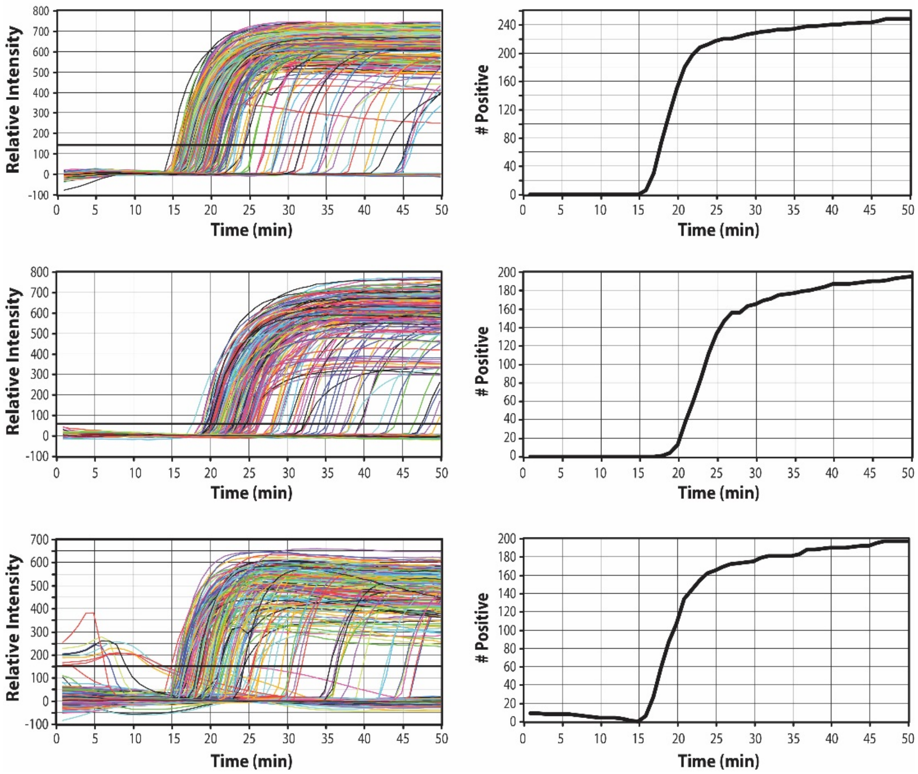
Relative Intensity
Time (min)
Positive

B)

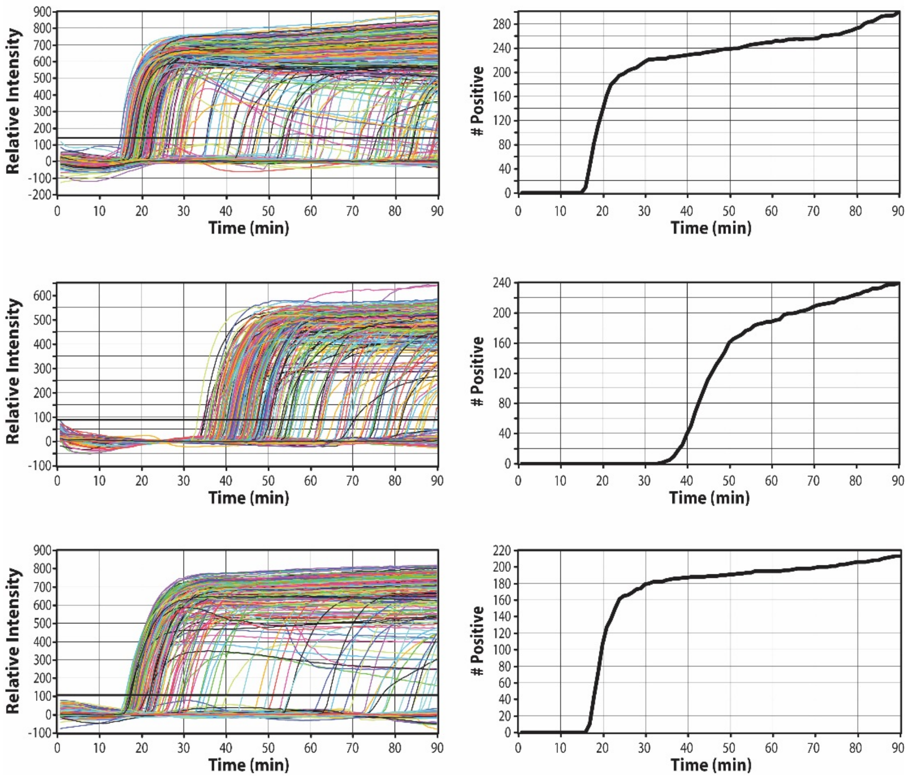
Relative Intensity
Time (min)
Positive

C)

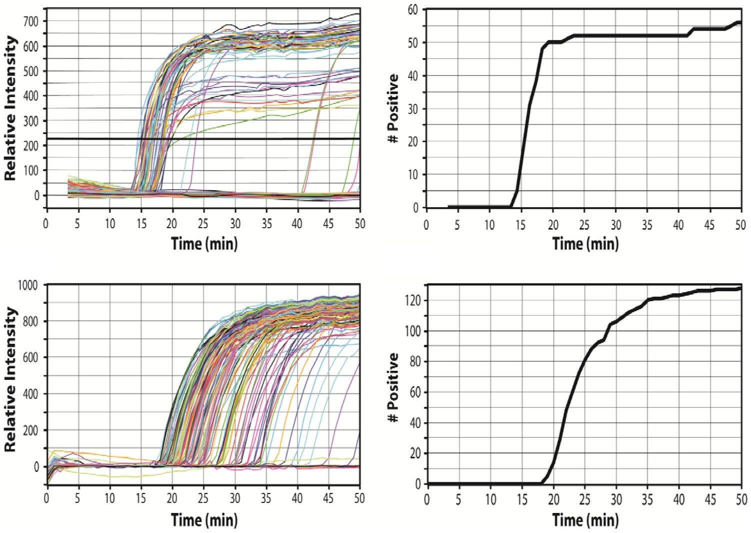

Figure S3. Examples of the data on real-time digital measurements of the cumulative fates and rates of the single template molecules. (A) when comparing the digitally optimized primer set (DOP) (top) and elongated BIP and FIP sets (DOP-LBIP and DOP-LFIP) (middle and bottom); (B) when comparing the digitally optimized primers sets with both loop primers (DOP) (top), with no loop primers (DOP-NL) (middle) and with no loop F primer only (DOP-NLF) (bottom); (C) when comparing two different enzymes mixtures, EM (top) and RtxBst (bottom).
